# Production of Fluconazole-Loaded Polymeric Micelles Using Membrane and Microfluidic Dispersion Devices

**DOI:** 10.3390/membranes6020029

**Published:** 2016-05-25

**Authors:** Yu Lu, Danial Chowdhury, Goran T. Vladisavljević, Konstantinos Koutroumanis, Stella Georgiadou

**Affiliations:** Chemical Engineering Department, Loughborough University, Loughborough, Leicestershire LE11 3TU, UK; y.lu2-11@student.lboro.ac.uk (Y.L.); D.S.Chowdhury-09@student.lboro.ac.uk (D.C.); K.Koutroumanis@lboro.ac.uk (K.K.); S.Georgiadou@lboro.ac.uk (S.G.)

**Keywords:** polymeric micelles, microfluidic co-flow, membrane contactor, fluconazole, drug-loaded micelles, diblock copolymers

## Abstract

Polymeric micelles with a controlled size in the range between 41 and 80 nm were prepared by injecting the organic phase through a microengineered nickel membrane or a tapered-end glass capillary into an aqueous phase. The organic phase was composed of 1 mg·mL^−1^ of PEG-*b*-PCL diblock copolymers with variable molecular weights, dissolved in tetrahydrofuran (THF) or acetone. The pore size of the membrane was 20 μm and the aqueous/organic phase volumetric flow rate ratio ranged from 1.5 to 10. Block copolymers were successfully synthesized with M_n_ ranging from ~9700 to 16,000 g·mol^−1^ and polymeric micelles were successfully produced from both devices. Micelles produced from the membrane device were smaller than those produced from the microfluidic device, due to the much smaller pore size compared with the orifice size in a co-flow device. The micelles were found to be relatively stable in terms of their size with an initial decrease in size attributed to evaporation of residual solvent rather than their structural disintegration. Fluconazole was loaded into the cores of micelles by injecting the organic phase composed of 0.5–2.5 mg·mL^−1^ fluconazole and 1.5 mg·mL^−1^ copolymer. The size of the drug-loaded micelles was found to be significantly larger than the size of empty micelles.

## 1. Introduction

Micellization is the process of forming oriented colloidal aggregates (micelles) that happens spontaneously in an aqueous solution of amphiphilic molecules (amphiphiles), composed of hydrophilic and hydrophobic portions, when its concentration exceeds the *critical micelle concentration* (CMC) at a certain temperature [1]. The hydrophilic segments (groups) are located at the surface of the micelles, whereas the hydrophobic segments form the hydrophobic core. Amphiphilic diblock copolymers are amphiphiles made up of two distinct homopolymer chains, hydrophobic and hydrophilic, linked by covalent bonds. The shape of polymeric micelles is believed to be spherical if the hydrophobic segment is longer than the hydrophilic segment; conversely various non-spherical structures, including rods and lamellae, may be formed when there is an increase in the length of the hydrophilic part [2]. Since the polymeric micelles with a diameter of 10–100 nm can be applied as nanoscale drug carriers, increasing studies on micellar drug delivery systems have been conducted using block copolymers with poly(ethylene glycol) (PEG) as a hydrophilic segment [3]. Hydrophobic segments of diblock copolymers applied for drug delivery are usually polyester or poly(amino acid) derivatives. Poly(lactic acid) (PLA), poly(ε-caprolactone) (PCL) and poly(glycolic acid) (PGA) are some of the most commonly used hydrophobic segments because of their biocompatible and biodegradable features [4].

Microfluidics is a research area focusing on transport phenomena in fluids at length scales of less than 1 mm and typically 1–500 μm [5]. Microfluidic technology has been extensively studied since the 1990s and applied especially to life and materials sciences and in the pharmaceutical industry. The benefit of small length scales and precise control of fluid flows was one of the main reasons that microfluidic technology attracted such great attention [6]. This technology provides the opportunity to conduct processes such as mixing of fluid streams; mass transfer operations; encapsulation; and droplet generation and manipulation (merging, splitting, and sorting) continuously with very low consumptions of materials and to integrate/combine many unit operations on a single chip [7]. Three major configurations of microfluidic mixing and drop generation units are co-flow, T-junction (cross-flow) and flow-focusing [8,9]. A variety of microfluidic devices have been used to produce nanoscale particles such as semiconductor quantum dots, metal colloids, liposomes, lipid nanotubes, polymeric, and composite nanoparticles [6,10,11,12].

Membrane technology has been widely applied in separation processes [13] and chemical/biochemical reactors [14]. However, in the past two decades microporous membranes are increasingly used for production of liquid–liquid, gas–liquid and solid–liquid dispersions, such as emulsions, gas dispersions and dispersions of micro/nano- particles [15,16]. In membrane dispersion processes, a membrane does not serve as a separation barrier, but a dispersion medium enabling controlled injection rates, uniform hydrodynamic conditions at the interface and higher production rates compared to microfluidic devices.

In this study, micelles composed of various amphiphilic diblock copolymers have been prepared by flash precipitation initiated by injection of the organic feed stream through a membrane into the water phase under controlled hydrodynamic conditions. The process is similar to membrane emulsification, except that the organic phase contains a water-miscible solvent, which diffuses into the water phase upon mixing causing copolymer molecules dissolved in the organic phase to self-assemble into micelles. In membrane emulsification, the organic phase contains a water-immiscible solvent or non-volatile organic liquid (oil).

The process was also investigated using a co-flow microfluidic device composed of two coaxial glass capillaries inserted into each other. The main objective was to analyze the effects of operating parameters on the size of micelles produced from both devices and to demonstrate encapsulation of the drug fluconazole within the micelles.

## 2. Experimental Section

### 2.1. Reagents and Materials

Poly(ethylene glycol) methyl ether (M_n_ ~5000 g·mol^−1^) (PEG), ε-caprolactone monomer 99% (ε-CL), tin(II) 2-ethylhexanoate 95% (Sn(Oct)_2_), and sodium silicotungstate were supplied by Sigma-Aldrich, Co Ltd, Gillingham, Dorset, UK. Toluene, extra dry grade, was purchased from Acros Organics, Geel, Belgium. PEG was dried by azeotropic distillation with toluene prior to the polymerization reaction. ε-CL was dried prior use by distillation under reduced pressure onto 3A molecular sieves. Tetrahydrofuran (THF) and acetone of analytical grade were purchased from Fischer Scientific (Loughborough, UK) and used without further purification. Fluconazole (pharmaceutical secondary standard, a molecular weight of 306.27 g·mol^−1^) was purchased from Sigma-Aldrich and used for drug loading experiments. Deionized water was obtained from a Millipore Synergy^®^ system (Ultrapure Water System, Merck Millipore, Darmstadt, Germany).

Polytetrafluoroethylene (PTFE) tubing supplied by Sigma-Aldrich was used for sample collection (inner diameter (I.D.) = 1.58 mm, outer diameter (O.D.) = 2.16 mm) and organic phase injection (I.D. = 0.8 mm, O.D. = 1.59 mm) in the microfluidic device. Polyethene (PE) tubing supplied from Smiths Medical Int’l Ltd, Kent, UK (I.D. = 0.86 mm, O.D. = 1.27 mm) was used for aqueous phase injection in the microfluidic device. Borosilicate round capillaries from Intracel Ltd, St Ives, UK (O.D. = 1.0 mm, I.D. = 0.58 mm) and borosilicate square capillaries from AIT Glass (O.D. = 1.5 mm, I.D. = 1.05 mm) were used for microfluidic device fabrication. The PTFE tubing (I.D. = 4.0 mm, O.D. = 6.36 mm) for organic phase injection in membrane experiments was purchased from Sigma-Aldrich.

### 2.2. Synthesis of Diblock Copolymers

One gram of PEG and 40% (*v*/*v*) ε-CL solution in toluene were dissolved in 20 mL of refluxing toluene under nitrogen atmosphere. The mole ratio of PEG to ε-CL in the reaction mixture was varied to produce a PEG:PCL ratio from 1:1.5 to 1:3.0. The polymerization was initiated by the addition of a 20% (*v*/*v*) solution of Sn(Oct)_2_ in toluene (0.75 *w*/*w*) and carried out at 110 °C under nitrogen atmosphere and constant stirring for 18 h. The PEG-*b*-PCL copolymer was isolated by precipitation in diethyl ether and dried under vacuum.

### 2.3. Fabrication of Co-Flow Microfluidic Device

**Figure 1:**
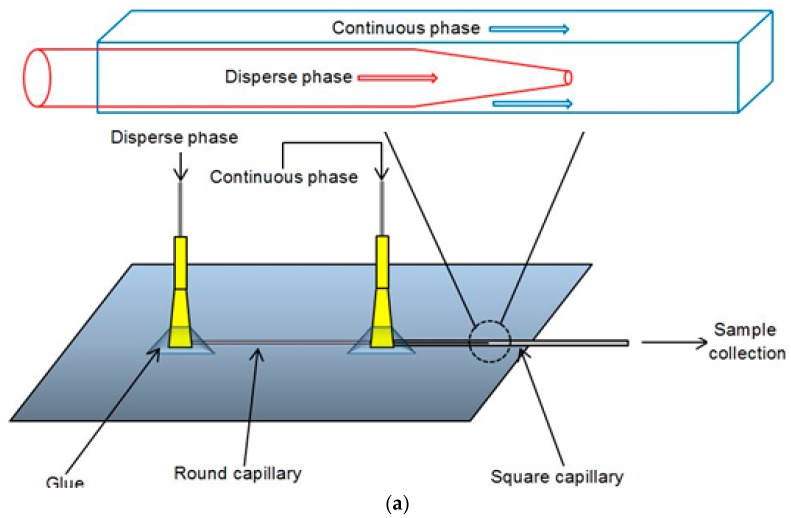

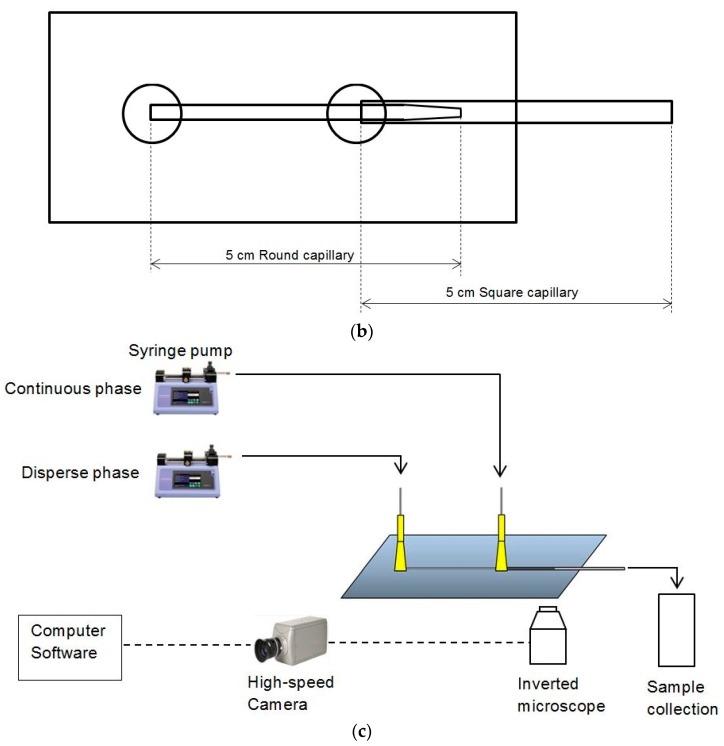
(**a**) Schematic diagram of a co-flow glass capillary device used in the study and a magnified schematic diagram of the co-flow region in the device; (**b**) top view of the device; and (**c**) schematic diagram of the experimental set-up for co-flow microfluidic experiments.

The co-flow microfluidic device (Figure 1) consisted of a 5 cm long tapered-end round capillary for supplying the organic phase, inserted in a 5 cm coaxial square capillary, which the aqueous phase flowed through. The round capillary was prepared by pulling apart a whole capillary using a Flaming-Brown type micropipette puller (P-97, Sutter Instruments, Novato, CA, USA) and then precisely rubbing the tip with sandpaper to a desired tip diameter. The tip was treated with 2-[methoxy(polyethylenoxy)propyl]trimethoxysilane to enhance the hydrophilicity of its surface. After flushing the round capillary with water and drying with compressed air, it was inserted into the square capillary, which had been glued and fixed onto a transparent glass plate. The glue used was two-component epoxy glue (ITW Devcon Ltd., Shannon, Ireland). Two needles (PrecisionGlide 305175, Becton Dickinson and Company, Franklin Lakes, NJ, USA), which were cut to remove the sharp tips, were positioned and glued, one at the end of the round capillary and another at the junction of the two capillaries. The device was tested by running water through each needle to ensure that no leaking or blocking was present.

### 2.4. Experiment Setup

#### 2.4.1. Experimental Setup for Microfluidic Device

Figure 1c shows the schematic diagram of the experimental set-up for co-flow microfluidic device experiments. Organic phase and aqueous phase were separately injected into the microfluidic device through tubing by syringes (SGE, Victoria, Australia), which were driven by syringe pumps (Harvard Apparatus; pump 11 Elite, Instech, Plymouth Meeting, PA, USA). This pump offers enhanced flow performance with high accuracy and smooth flow. The microfluidic device was placed on the stage of an inverted microscope (GX Microscope XDS-3, GX Optical, Suffolk, UK), which was connected to a high speed camera (Phantom v5.1, Vision Research, Wayne, NJ, USA) to observe and record live images. A tip size of the microfluidic device was 160 μm.

#### 2.4.2. Experimental Set-Up for Membrane Device

**Figure 2:**
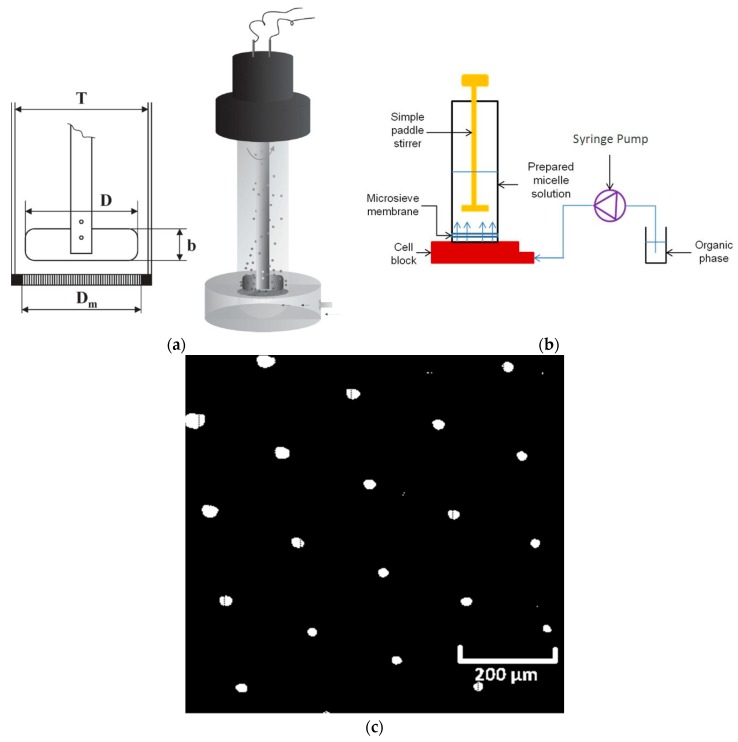
(**a**) Schematic diagram of stirred cell with paddle stirrer above a flat disc membrane; Diameter of stirring blade, D = 32 mm, width of stirring blade, b = 12 mm, diameter of stirred cell, T = 40 mm, and effective diameter of membrane, D_m_ = 33 mm. (**b**) Schematic diagram of the experimental set-up for membrane device. (**c**) A micrograph of the membrane surface showing cylindrical pores with constant pore to pore spacing.

The membrane device used in this work was a stirred cell with a flat disc membrane fitted under the paddle blade stirrer, as shown in Figure 2a. Similar to the microfluidic device, the organic phase was injected into the system through PTFE tubing using a syringe pump. However, unlike the microfluidic device, the aqueous phase was fixed in the cell, where a paddle blade stirrer was placed in, before injecting organic phase. The membrane used was a nickel membrane with a regular hexagonal pore array containing cylindrical pores with a diameter of 20 μm, spaced 200 μm from each other. Figure 2c is a magnified image of the membrane surface taken by an optical microscope. Both stirred cell and membranes were supplied by Micropore Technologies Ltd. (Hatton, Derbyshire, UK). The agitator was driven by a 24 V DC motor (PR 3060, GWInstek, New Tapei City, Taiwan) and the paddle rotation speed was controlled by the applied voltage.

### 2.5. Experimental Procedure

#### 2.5.1. Experimental Procedure of Microfluidic Experiments

Organic phase solution was prepared by dissolving the certain amount of copolymer (PEG-*b*-PCL (A), PEG-*b*-PCL (B) or PEG-*b*-PCL (C), weighed by electronic weighing balance) into an appropriate amount of organic solvent (THF or acetone). The concentration of copolymer in the organic phase was 1 mg·mL^−1^. The solution was sonicated in an ultrasonic bath (Fisher Scientific, Loughborough, UK) to ensure that the copolymer was dissolved completely. Syringes with volumes of 5 and 10 mL were used for organic and water phase injection, respectively. The water phase was first injected at fixed flowrate of 5 mL·h^−1^ and the organic phase was supplied at 0.1 to 3.33 mL·h^−1^, corresponding to Q_a_/Q_o_ ratios of 50 to 1.5. Then the organic phase was kept to 0.6 mL·h^−1^ with water phase flow rate changing from 0.1 to 15 mL·h^−1^. For the preparation of drug-loaded micelles, the organic phase was made up of 0.6 mg·mL^−1^ of fluconazole and 1.5 mg·mL^−1^ of the copolymer in acetone. An ultrasonic bath was used for dissolving fluconazole and copolymer.

#### 2.5.2. Experimental Procedure of Membrane Experiments

The organic phase for both drug-free and drug-loaded experiments was prepared according to the procedure explained in Section 2.5.1. The stirred cell (the Dispersion Cell) was filled with 25 mL of deionized water and the stirring speed was set to 700 rpm. Then, 5 mL of the organic phase was injected through the membrane for ~31 s at a flow rate of 9.6 mL·min^−1^ to achieve a final aqueous to organic phase volume ratio of 5. The micelle suspension was kept under stirring for 1 min after organic phase injection. After each experiment, the membrane was sonicated in THF for 1 h. For drug-loaded micelles, the organic phase was made up of 0.5, 1.0 or 2.5 mg·mL^−1^ of fluconazole and 1.0 mg·mL^−1^ of the copolymer in acetone. The Zeta potential was measured for drug-loaded and drug-free micelles for all three copolymers.

### 2.6. Process Reproducibility Experiments

The experiments conducted under a certain set of conditions were repeated three times in order to estimate reproducibility of the fabrication process. The conditions for microfluidic experiments were: Q_a_ = 5 mL·h^−1^, Q_o_ = 1.25 mL·h^−1^ (Q_a_/Q_o_ = 4) and the conditions for membrane device were: V_o_ = 5 mL, V_w_ = 25 mL. The organic phase solution for both devices was 1 mg·mL^−1^ of the copolymer PEG-*b*-PCL (B) dissolved in acetone.

### 2.7. Micelle Size Measurement

The size of the micelles was measured using Delsa™Nano C particle analyser from Beckman Coulter Inc., Brea, CA, USA. This equipment uses photon correlation spectroscopy (PCS), which determines particle size by measuring the rate of fluctuations in laser light intensity scattered by particles as they diffuse through a fluid. The samples were stored under vacuum to achieve a complete evaporation of acetone/THF and then transferred to a measuring cuvette (Fisherbrand™ macro, PS, 12.5 mm × 12.5 mm × 40 mm, Fisher Scientific, Loughborough, UK) and placed into the machine. A refractive index of 1.3300, a viscosity index of 0.89 and dielectric constant of 78.3 were used. All samples were measured in 3 runs, with each run taking approximately 4 min. The distribution data and mean size for each run were automatically produced from the software of the particle analyzer. Average size values for 3 runs were then calculated and recorded.

### 2.8. Zeta Potential Measurement

The Delsa™Nano C was also used for the measurements of the zeta potential. For this, an alternative electrophoretic light scattering mode was used with a fixed applied voltage of 60 V.

## 3. Results and Discussion

### 3.1. Polymer Characterization

Gel Permeation Chromatography (GPC) was used to determine the molecular weight, polydispersity index (M_w_/M_n_), and hydrophilic fractions (f) of the synthetized copolymers. GPC analysis was performed using an Agilent 1100 High-performance Liquid Chromatography (HPLC) System (Agilent Technologies, Santa Clara, CA, USA) equipped with a refractive index detector (G1362A). Analysis was performed on an Agilent PLgel MIXED-C column (Agilent Technologies, Santa Clara, CA, USA), 5 μm, temperature was 30 °C. Calibration was performed using polystyrene standards with a narrow molecular weight distribution (EasiVials PS-M, Agilent Technologies, Santa Clara, CA, USA). The results are listed in Table 1 and include both number-average and weight-average values. Hydrophilic fraction is the average fraction of the hydrophilic block (PEG) in the copolymer.

**Figure 3:**
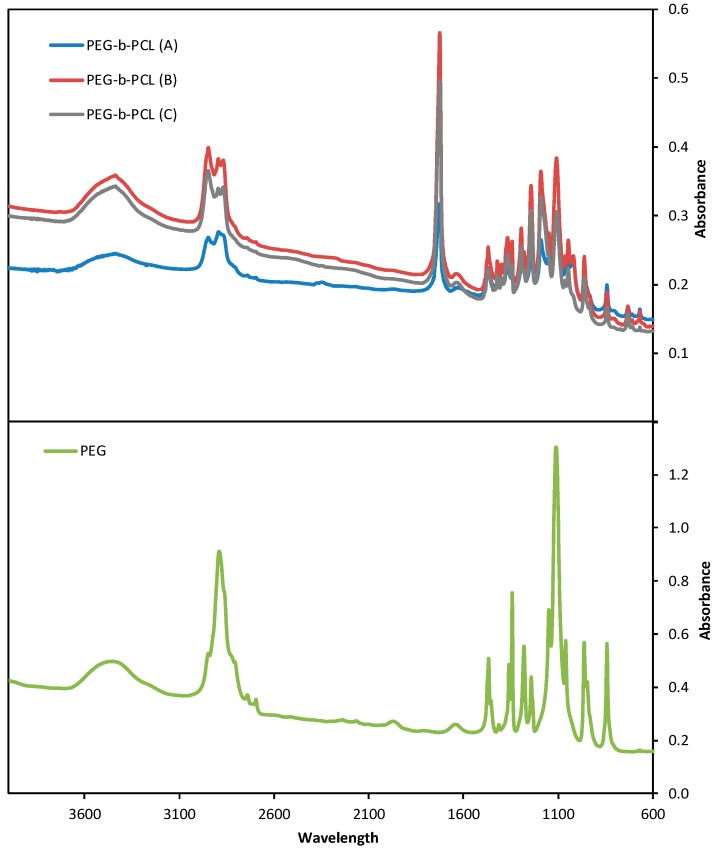
Fourier Transformed Infrared Spectroscopy (FTIR) spectra for Poly(ethylene glycol) (PEG) and the synthesized Poly(ethylene glycol)-b-poly(ε-caprolactone) copolymers.

Fourier Transformed Infrared Spectroscopy (FTIR) spectra are shown in Figure 3. FTIR spectra were obtained using a Shimadzu FTIR-8400S spectrometer. A small amount of each material was mixed with KBr and compressed to tablets. The IR spectra of these tablets were obtained in absorbance mode and in the spectral region of 600–4000 cm^−1^ using a resolution of 4 cm^−1^ and 64 co-added scans. All materials show characteristic absorptions for PEG, the C-O-C etheric bond bending vibration at 1109 cm^−1^ and the absorptions at 842 and 1333 cm^−1^, attributed to PEG crystalline regions. On the PEG-*b*-PCL spectra, new absorptions emerge: one at 1724 cm^−1^ is attributed to stretching of the esteric carbonyl, while the two at 2935 and 729 cm^−1^ are due to C-H bond stretching in the PCL block. Absorptions attributed to the PCL block increase in intensity from PEG-*b*-PCL (A) to PEG-*b*-PCL (C) to PEG-*b*-PCL (B), as the molecular weight of the hydrophobic block increases, respectively.

**Figure 4:**
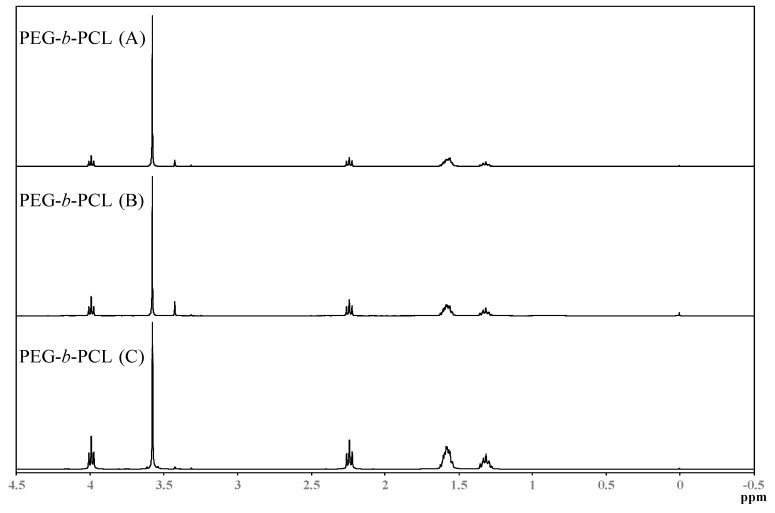
Nuclear Magnetic Resonance Spectroscopy (1H-NMR) spectra for the synthesized PEG-*b*-PCL copolymers.

Spectra from Nuclear Magnetic Resonance Spectroscopy (1H-NMR) are shown in Figure 4. NMR spectra were obtained on a JEOL-ECS-400 NMR spectrometer operating at 400.13 MHz for protons, employing a 5 mm high-resolution broad-band gradients probe. Spectra were recorded using the “single-pulse” pulse program with P90 = 10.4 μs covering a sweep width 25.0 ppm (8278 Hz) with 32,000 time domain data points giving an acquisition time of 3.95 s, Fourier transformed using 64,000 data points and referenced to an internal TMS standard at 0.0 ppm. Absorbencies at 4.0 and 2.3 ppm are due to protons in the PCL block, while the absorbance at 3.3 ppm is due to the three protons in the methoxy terminal-group of PEG. The absorbance of the PCL block increases in intensity when the hydrophobic block increases, respectively.

### 3.2. Experimental Images from Microfluidic Device

**Figure 5:**
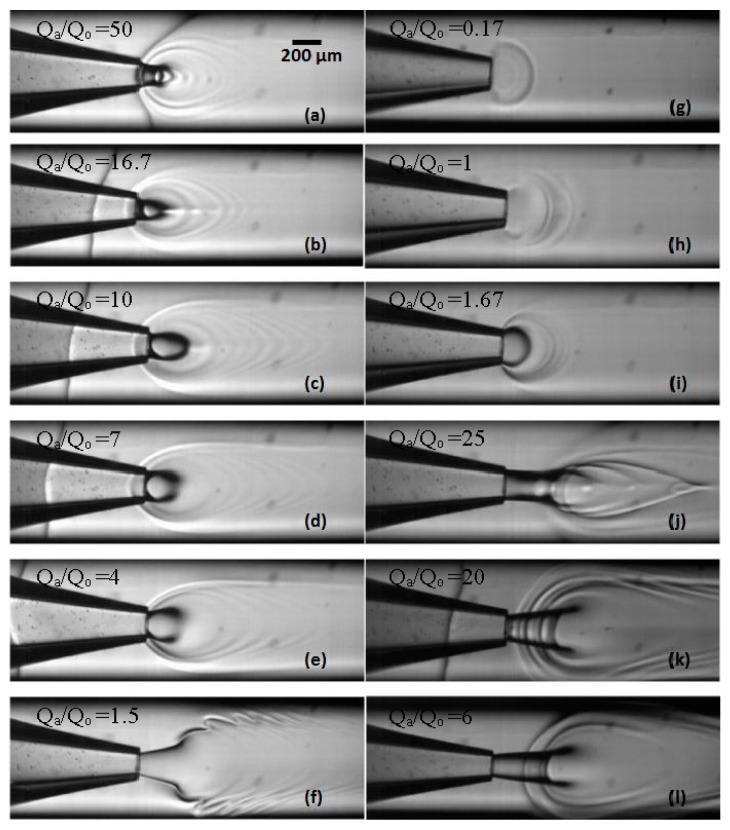
Experimental images using a co-flow device (tip size = 160 μm) from a high speed camera with varying aqueous phase flow rates (Q_a_) and organic phase flow rates (Q_o_): (**a**) Q_a_ = 5 mL·h^−1^, Q_o_ = 0.1 mL·h^−1^, Q_a_/Q_o_ = 50; (**b**) Q_a_ = 5 mL·h^−1^, Q_o_ = 0.3 mL·h^−1^, Q_a_/Q_o_ = 16.7; (**c**) Q_a_ = 5 mL·h^−1^, Q_o_ = 0.5 mL·h^−1^, Q_a_/Q_o_ = 10; (**d**) Q_a_ = 5 mL·h^−1^, Q_o_ = 0.71 mL·h^−1^, Q_a_/Q_o_ = 7; (**e**) Q_a_ = 5 mL·h^−1^, Q_o_ = 1.25 mL·h^−1^, Q_a_/Q_o_ = 4; (**f**) Q_a_ = 5 mL·h^−1^, Q_o_ = 3.33 mL·h^−1^, Q_a_/Q_o_ = 1.5; (**g**) Q_a_ = 0.1 mL·h^−1^, Q_o_ = 0.6 mL·h^−1^, Q_a_/Q_o_ = 0.17; (**h**) Q_a_ = 0.6 mL·h^−1^, Q_o_ = 0.6 mL·h^−1^, Q_a_/Q_o_ = 1; (**i**) Q_a_ = 1 mL·h^−1^, Q_o_ = 0.6 mL·h^−1^, Q_a_/Q_o_ = 1.67; (**j**) Q_a_ = 15 mL·h^−1^, Q_o_ = 0.6 mL·h^−1^, Q_a_/Q_o_ = 25; (**k**) Q_a_ = 10 mL·h^−1^, Q_o_ = 3.33 mL·h^−1^, Q_a_/Q_o_ = 3; and (**l**) Q_a_ = 20 mL·h^−1^, Q_o_ = 3.33 mL·h^−1^, Q_a_/Q_o_ = 6. The scale bar in Figure 5a applies to all figures from Figure 5a–l. The organic solvent was tetrahydrofuran (THF).

Live images were recorded by the high speed camera and analyzed by the computer software Image J (v1.46). Figure 5 shows the images recorded under different phase flow rates of the aqueous (water) and organic phase. From Figure 5a–f, the volumetric flow rate of aqueous phase Q_a_ was fixed to 5 mL·h^−1^ and Q_o_ varied from 0.1 to 3.33 mL·h^−1^ to give Q_a_/Q_o_ values of 50, 16.67, 10, 7, 4 and 1.5, respectively. From Figure 5g–j, Q_o_ was fixed to 0.6 mL·h^−1^ and Q_a_ varied from 0.1 to 15 mL·h^−1^ to give Q_a_/Q_o_ values of 0.17, 1, 1.67 and 25, respectively. In both Figure 5k,l, Q_o_ was set to 3.33 mL·h^−1^ and Q_a_ was 10 or 20 mL·h^−1^ to achieve Q_a_/Q_o_ values of 3 or 6. The shape of organic/aqueous interface strongly depends on the flow rates of the two phases. At low flow rates, the interface is hemispherical and resembles the shape observed during generation of droplets in the dripping regime [17,18]. In droplet microfluidics, however, the organic phase eventually detaches from the tip and forms an isolated droplet in the aqueous phase, whereas in this case, the droplet does not grow on the tip, since THF and water are miscible in all proportions and the rate of transfer of THF to the interface by the pump is in equilibrium with the rate of transfer of THF away from the interface due to diffusion into the aqueous phase. The existence of curved interface is a result of the transient interfacial tension, the Korteweg stress, which occurs when two miscible fluids are suddenly put into contact [19]. The transient interfacial tension is given by: $\sigma =k\Delta C^{2}/\delta$, where ΔC is the change in concentration over the transition zone between two miscible fluids, δ is the thickness of the transition zone, and k is the proportionality constant. If the organic phase is not injected continuously, the transient tension would decrease rapidly in proportion to $\sqrt{D/t}$, where D is the diffusion coefficient and t is the interfacial age. However, because both liquids flow continuously, the Korteweg stress maintains a constant value over time at any location on the interface.

At low organic phase flow rates, Figure 5a–c, the liquid–liquid interface is sharp over the whole jet boundary, but at higher organic phase flow rates, it becomes blurry (Figure 5e) or even completely disappears (Figure 5g–i) on the front side of the jet. It can be explained by high THF concentration in the central region of the capillary, predicted by numerical modeling [11], which leads to low concentration gradient, ΔC, and negligible Korteweg stress on the front side of the jet. In most cases, the formed micelles are concentrically clustered around the interface (see Figure 5a–e), due to capillary waves formed on the surface of a drop under the influence of external fluid flow [20]. “Viscous fingering” can be seen in Figure 5f, which occurs due to non-uniform penetration arising when a less viscous fluid is injected at high relative velocity into a more viscous one. At 293 K, the viscosities of THF and water are 0.63 and 1 mPas, respectively, supporting this assumption. Viscous fingering did not occur in Figure 5k,l, although the flow rate of the organic phase was exactly the same as in Figure 5f, probably because Q_a_ was higher than in Figure 5f, and the difference in velocity between the two streams was not sufficiently high.

Due to the 3D geometry of the capillaries, the aqueous phase fully surrounds the organic phase and de-wets it from the walls of the outer capillary. Since the micelles are formed at the liquid–liquid interface, which is displaced from the walls, the deposition of the micelles onto the walls should be less pronounced compared with 2D polydimethylsiloxane microfluidic devices. However, a limited amount of deposited nanoparticles are visible in Figure 5a–d,k.

By increasing the water flow rate from 1 to 15 mL·h^−1^ at a constant organic phase flow rate of 0.6 mL·h^−1^, the interface is stretched from a hemisphere (Figure 5i) to an extended cylinder (Figure 5j), because the shear force acting on the interface from the surrounding aqueous stream overcomes the transient interfacial tension. It is interesting to note that the interface has entirely different shapes in Figure 5f,i, although the flow rate ratio was very similar. However, when the flow rates of both fluids are increased at constant flow rate ratio, it has more impact on the injection velocity of the organic phase than on the aqueous phase velocity. As a result, in Figure 5f the velocity of the organic phase in the orifice significantly surpasses the velocity of the aqueous phase and in Figure 5i both streams have a similar velocity.

### 3.3. Parameters Affecting the Diameter of Polymeric Micelles

#### 3.3.1. Copolymer Type

Table 2 shows the mean size of polymeric micelles from three different copolymers, two producing techniques, and two organic solvents. Polydispersity index (PDI) is a measure of broadness of particle size distribution. It can be seen that no significant difference in the micelle size was observed using different copolymers.

#### 3.3.2. Organic Solvent

Even though organic solvent was evaporated under vacuum, the solvents may still remain traceable in the final sample, so THF and acetone were chosen because of their low toxicity and good fluconazole (only in acetone) and PEG-*b*-PCL solubility [21]. From Table 2 it can be concluded that from both microfluidic and membrane preparation techniques and for all copolymers the size of micelles when using acetone as solvent was larger than using THF, even though the difference was minor. Laouini *et al.* [21] obtained larger micelles when using THF but found the diameter of micelles were largely unaffected by the solvent type. Such differences are most likely attributable to different solubility of the polymer in water–THF and water–acetone mixtures and different viscosities of the two solvents.

#### 3.3.3. Micelles Preparation Techniques

**Figure 6:**
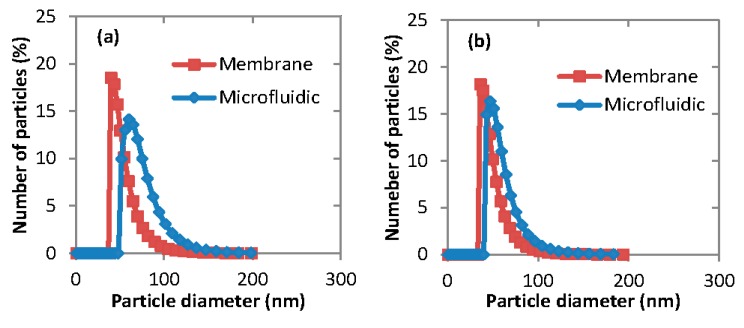

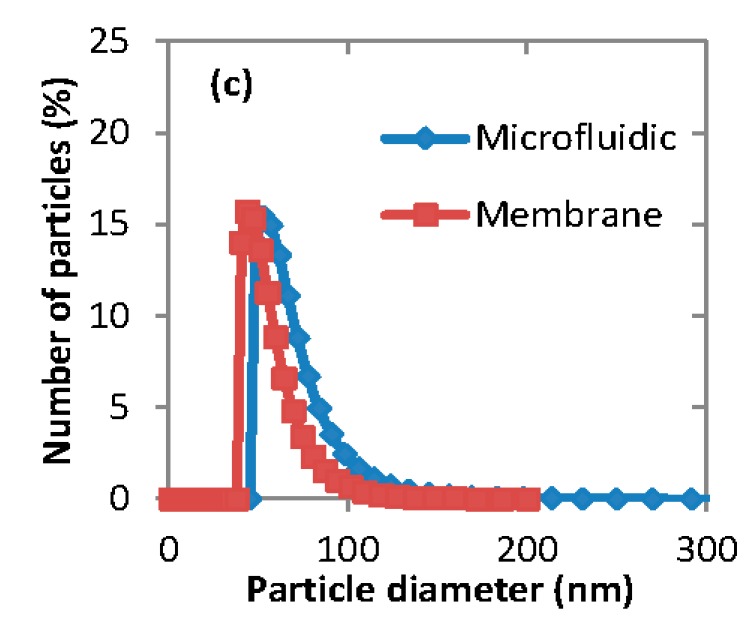
The particle size distribution of drug-free micelles produced using membrane and microfluidic techniques from different copolymers: (**a**) PEG-*b*-PCL (A); (**b**) PEG-*b*-PCL (B); and (**c**) PEG-*b*-PCL (C). The organic phase was 1 mg·mL^−1^ PEG-*b*-PCL in acetone.

As shown in Table 3 and Figure 6, drug-free micelles prepared in the Dispersion Cell were smaller than the micelles prepared using microfluidic device. It can be explained by the fact that in the Dispersion Cell, the organic phase was injected through a 20 μm pore-sized membrane, while in the microfluidic device the dispersed phase was injected through an orifice with a diameter of 160 μm. The same trend was observed when fluconazole was loaded into the hydrophobic core of the micelles, with even more significant difference between the membrane and microfluidic device. For example, the size of fluconazole-loaded micelles produced in the membrane device was 49–63 nm, depending of the type of the copolymer used (Table 2). On the other hand, the size of fluconazole-loaded micelles produced in the microfluidic device was 84–112 nm, which is a significant increase in the micelle size by 71%–78%.

Experiments in the Dispersion Cell have also been done with direct injection of the organic phase into the aqueous phase without using penetration through the membrane to reveal the effect of membrane at the same stirrer speed of 700 rpm, injection rate of the organic phase of 9.62 mL·min^−1^ and copolymer concentration in acetone of 1 mg·mL^−1^. As shown in Table 3, for all three copolymers investigated, the size of micelles produced without injection of the organic phase through the membrane was significantly larger than the size of micelles produced by membrane permeation. It is clear that the micelle size can be controlled to a certain extent by hydrodynamic conditions that can be accurately controlled in the membrane device. Similar results were obtained for polymeric micelles [21] and liposomes [22] using membranes with different pore sizes. In the absence of membrane, the organic phase is injected through a single hole at the bottom of the stirred cell, which means that organic solvent concentration in the aqueous phase is non-uniform on the micro-scale, with a very high concentration near the injection hole and low concentration elsewhere. In the case of membrane injection, the organic phase stream is split into numerous micro-jets, which come into contact with an aqueous phase. Since the pores are regularly arranged over the whole membrane surface, it leads to uniform mixing on the micro-scale without any bulk mixing.

**Figure 7:**
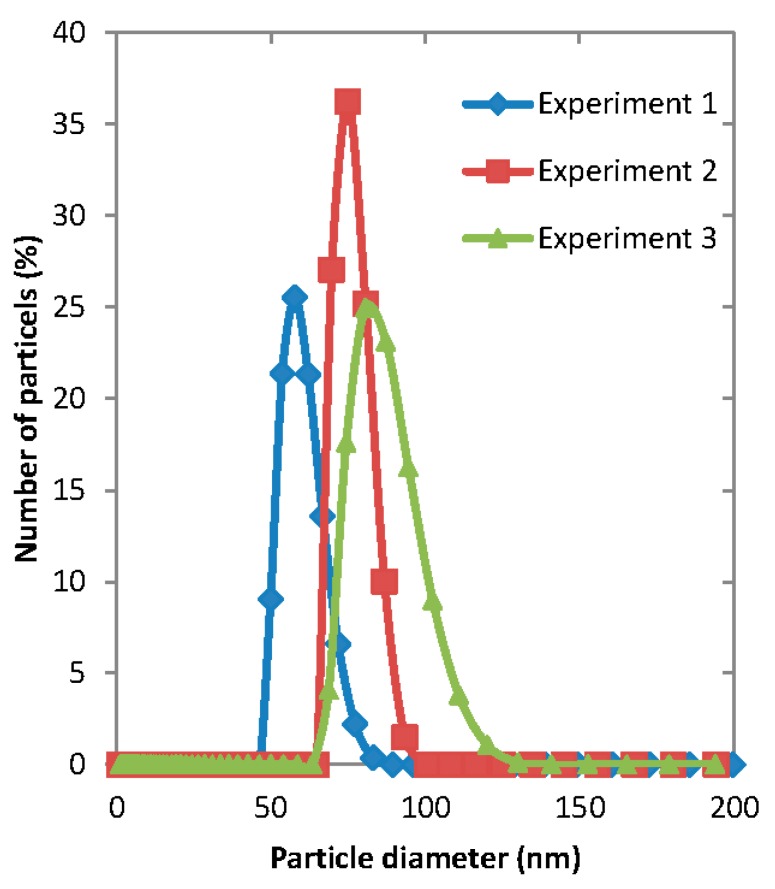
The particle size distribution of polymeric micelles prepared using Dispersion Cell without membrane for three repeated experiments performed under the same conditions. The organic phase was 1 mg·mL^−1^ of PEG-*b*-PCL (A) disolved in acetone, the total volume of the organic phase injected was 5 mL and the volume of the aqueous phase in the cell was 25 mL.

Another important consequence of direct solvent injection is that the particle size distribution was unpredictable and reproducibility of the experiment was extremely low, as illustrated in Figure 7. This indicates that the use of a fixed pore size membrane provides essential consistency to the system and allows control over particle sizes. Reproducibility of stirred cell with membrane and microfluidic device is discussed in Section 3.6.

#### 3.3.4. Aqueous/Organic Phase ratio

**Figure 8:**
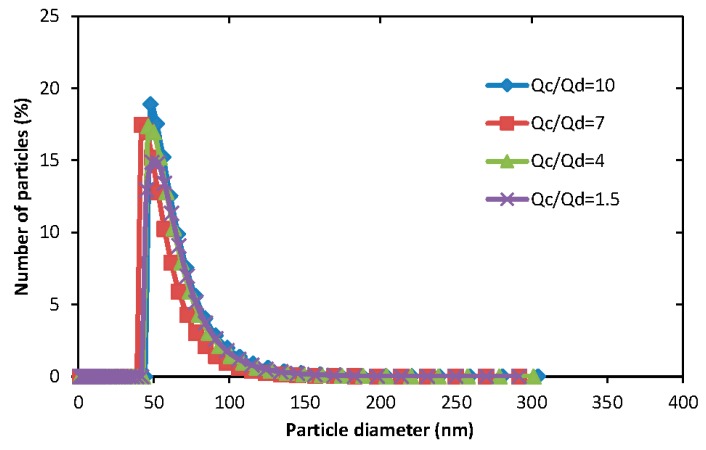
The particle size distribution of micelles generated from the same co-flow microfluidic device with an orifice diameter of 160 μm at different aqueous/organic flow rates ratios, Q_a_/Q_o_. Aqueous phase flow rates Q_a_ were fixed to 5 mL·h^−1^ for all four experiments. The organic phase was composed of 1 mg·mL^−1^ of PEG-*b*-PCL (B) dissolved in acetone.

Figure 8 shows the particle size distribution for micelles generated from the same co-flow microfluidic device under different aqueous/organic volume ratio. These illustrate that there are no significant differences of particles diameters among different Q_a_/Q_o_ values. Laouini *et al.* [21] found that the diameter of micelles generated from membrane device uniformly decreased when increasing the aqueous/organic phase ratio.

### 3.4. Fluconazole-Loaded Micelles

**Figure 9:**
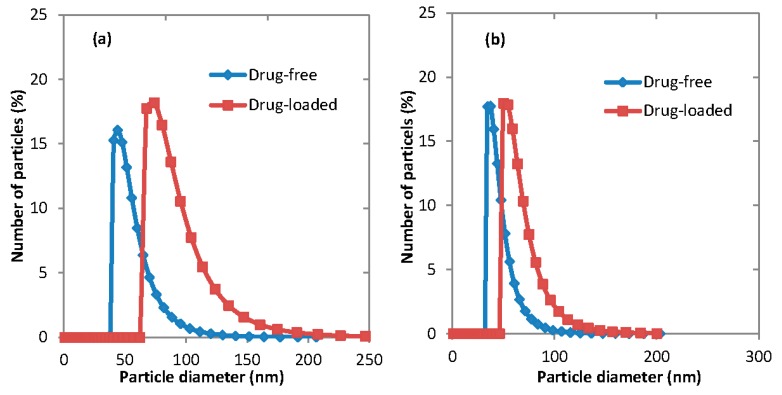
The particle size distribution of empty and drug-loaded micelles prepared using two different devices: (**a**) Microfludic; and (**b**) Membrane. For drug-free micelles, the organic phase concentration was 1 mg·mL^−1^ PEG-*b*-PCL (B) in acetone. For drug-loaded experiments, the organic phase was a mixture of 0.6 mg·mL^−1^ fluconazole and 1.5 mg·mL^−1^ PEG-*b*-PCL (B) in acetone. The aqueous/organic phase ratio was Q_a_/Q_o_ = 0.71.

Fluconazole is a triazole antifungal agent that is clinically widely used due to its advantages of excellent bioavailability and low toxicity [23]. Fluconazole was added into organic phase solution to prepare micelles with their hydrophobic cores loaded with the drug. Because of the low solubility of fluconazole in THF, only acetone was used as the organic solvent for organic phase. The mean diameters of fluconazole-loaded micelles are provided in Table 2. Figure 9 shows the effect of drug loading on the particle size distribution of micelles generated using microfluidic and membrane devices. It can be seen that under all process conditions, drug-loaded micelles were larger than drug-free micelles, which agrees with the results of similar studies [24].

#### 3.4.1. Effect of Fluconazole Concentration in Organic Phase on Micelle Size

The impact of varied concentrations of fluconazole in the organic phase upon the size of the micelles was investigated using only the membrane device. Table 4 shows the variation of the average micelle size when the drug concentration in the organic phase increased from 0.5 to 2.5 mg·mL^−1^ at the fixed concentration of PEG-*b*-PCL (B) of 1 mg·mL^−1^. It is clear that increasing the drug concentration in the organic phase results in an increase in the micelle diameter. This can be attributed to the presence of more drug that is loaded into the micelle, and demonstrates that the micelles are able to swell to accommodate more drug—increasing the micelle size. Further investigation could establish whether there is a maximum drug-loading capacity of PEG-*b*-PCL, where higher concentrations of drug are merely precipitated into the bulk.

#### 3.4.2. Effect of Fluconazole Concentration in Organic Phase on Encapsulation

**Figure 10:**
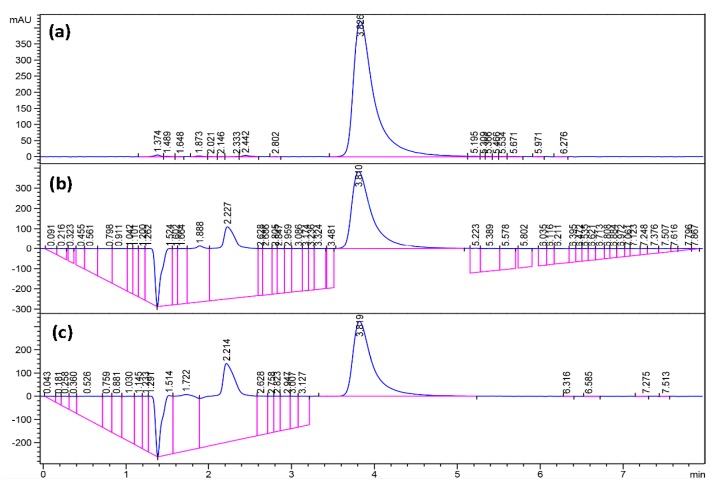
High-performance liquid chromatography (HPLC) analysis of: (**a**) known concentration of 0.5 mg·mL^−1^ of fluconazole for calibration; (**b**) supernatant of drug-loaded micelles using PEG-*b*-PCL (B) after 48 h; and (**c**) supernatant of drug-loaded micelles using PEG-*b*-PCL (C) after 48 h.

The amount of encapsulation of fluconazole from the organic phase was investigated from micelles produced from the membrane device. HPLC analysis was performed using an Agilent 1100 HPLC System and a Supelco Ascentis C18 column, 5 μ, 150 mm × 4.6 mm. The mobile phase was methanol:water (97:3, *v*/*v*), and the analyte was detected at 292 nm. The flow rate of the mobile phase was 1.2 mL·min^−1^ and the column temperature was 30 °C. Quantification of fluconazole content was based on a calibration curve created by assaying samples with concentrations of 0.1, 0.2, 0.25, 0.3 and 0.5 mg·mL^−1^ of fluconazole. Drug-loaded micelles of 0.4 mg·mL^−1^ per 1 mg·mL^−1^ PEG-*b*-PCL were centrifuged using an OptimaTM Ultracentrifuge (Beckman Coulter Inc, California, USA) at 50,000 rpm for 50 min at +4 °C to separate micelles from non-encapsulated drug. The supernatant non-encapsulated drug was then assayed for fluconazole content using HPLC. Representative results are shown in Figure 10—where fluconazole peak is visible beyond 3.5 min for each graph. A mass balance was performed to determine the encapsulated drug.

Table 5 shows the drug encapsulation over time when using the membrane device with polymers PEG-*b*-PCL (B) and PEG-*b*-PCL (C) to produce micelles. The results demonstrate the encapsulation of drug, and the release of drug after a period of 48 h. On Day 0, fluconazole causes the micelles to swell and is encapsulated within the micelles as displayed by results for both PEG-*b*-PCL (B) and PEG-*b*-PCL (C). On Day 2, the concentration of fluconazole decreases significantly for both PEG-*b*-PCL (B) and PEG-*b*-PCL (C), indicating a release of fluconazole into the bulk liquid.

### 3.5. Micelles Stability

**Figure 11:**
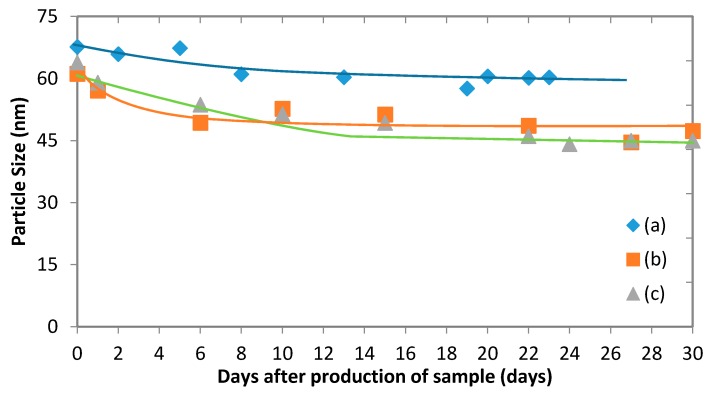
(**a**) The change in average particle size of micelles during storage: (**a**) Drug-free micelles produced in microfluidic device at Q_a_ = 5 mL·h^−1^ and Q_o_ = 0.71 mL·h^−1^. The organic phase was 1 mg·mL^−1^ PEG-*b*-PCL (B) in THF; (**b**) Drug-loaded micelles produced using membrane device. The fluconazole content in the organic phase was 0.4 mg·mL^−1^ per 1 mg·mL^−1^ PEG-*b*-PCL (A); (**c**) Drug-loaded micelles produced using membrane device. The fluconazole content in the organic phase was 0.4 mg·mL^−1^ per 1 mg·mL^−1^ PEG-*b*-PCL (B).

The stability of polymeric micelles is one of the most challenging obstacles of applying them in drug delivery systems since they are still a physically assembled structure. Figure 11 illustrates the change of the average particle size of drug-loaded and empty micelles, which occurs during storage. It shows that while there is an initial drop in the micelle size, it tends to remain steady after just over a week. The stability of polymeric micelles is affected by many factors such as polymer type, concentration, and molecular weight [25]. Kim *et al.* [25] stated that the micelle stability depends on the polymer concentration, which should be higher than the CMC to ensure the micelles are not disintegrated into unimers. The stability of micelles can be directly linked to *E*_bend_, which is the elastic energy of the membrane interface of micelles [26]. Two models of calculating *E*_bend_ were proposed: Helfrich model [27] and Hyde model [28] (the surfactant parameter model). In Hyde’s model, *E*_bend_ is expressed depending on the surfactant parameter of the amphiphilic molecule (p), which depends on the polar head area, the volume and the effective length of the hydrocarbon chain of the amphiphilic chain, and the characteristic value of p in absence of bending stresses (p_0_). Here, no evidence has shown that the micelles began to disintegrate to unimers during the testing period. It is likely that the initial drop in size can be attributed to solvent that remains in the micelle after vacuum evaporation. Indeed, small amounts of THF and acetone can remain in the sample and evaporate over time, causing the micelles to shrink. In the case of empty micelles, the initial drop is less significant than with drug-loaded micelles. This suggests that there might be a combined effect of evaporation of solvent and release of fluconazole into the bulk liquid [29].

#### Zeta Potential

Zeta potential is a measure of the electrical potential between the bulk fluid and the interface of a particle. A largely negative or positive value indicates high electrostatic forces leading to micelle stability [30].

The zeta potential was measured for fluconazole-loaded and empty micelles produced from membrane experiments (Table 6). The existence of a negative zeta potential can be associated to the carboxylic group contained on PLC chains [21]. Given that good stability of the micelles was observed (Figure 11), these low zeta potential values suggest a steric stability due to the PEG-*b*-PCL copolymers. After loading fluconazole, a lower absolute zeta potential was observed. This can be attributed to some of the drug adsorbing to the surface of the micelle and shielding the negative charge. Given the relatively high stability of the micelles found in Figure 11, it can be concluded once again that the micelles exhibit a strong steric stability despite the presence of a drug.

### 3.6. Process Reproducibility

**Figure 12:**
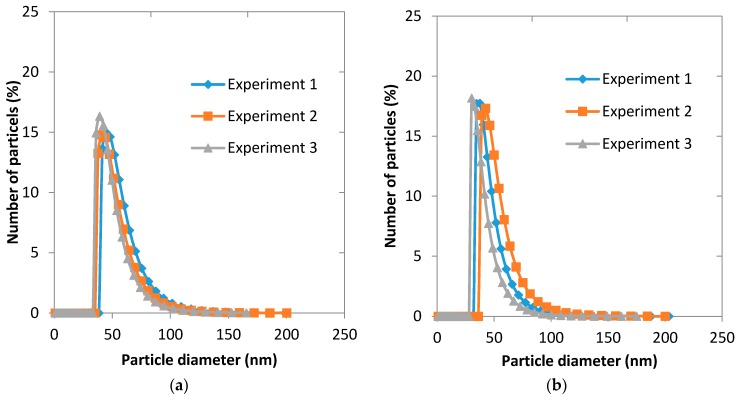
Reproducibility of experiments: (**a**) co-flow glass capillary microfluidic device; and (**b**) membrane device.

Reproducibility of the particle size distribution of micelles was estimated by repeating one membrane and one microfluidic experiment three times. For microfluidic experiment, the organic phase was 1 mg·mL^−1^ of the copolymer dissolved in acetone and Q_a_/Q_o_ = 7. For membrane experiment, 5 mL of the organic phase composed of 1 mg·mL^−1^ of the copolymer dissolved in acetone was injected into 25 mL of water. It can be seen from Figure 12 that the both preparation techniques gave a very good reproducibility in terms of the average micelle size produced under the same process conditions.

## 4. Conclusions

A novel co-flow glass capillary microfluidic device with 160 μm orifice size and a stirred cell with 20 μm pore-sized membrane were used to produce polymeric micelles composed of hydrophobic poly(caprolactone) (PCL) core and hydrophilic poly(ethylene) glycol (PEG) shell. Block copolymers composed of PEG and PCL with three different PEG/ε-CL mole ratios and molecular weights were successfully synthesized and used as building blocks for the preparation of both empty and fluconazole-loaded micelles. The organic phase was copolymer dissolved in THF or acetone for empty micelles preparation and a mixture of copolymer and fluconazole dissolved in acetone for drug-loaded experiments. It was found that the micelles produced from the membrane device were smaller than those produced from microfluidic device and the aqueous/organic volumetric ratio was found to have no noticeable effect on the diameter of the micelles. After loading fluconazole in the core, the diameter of micelles increased as expected for all copolymers tested and both preparation techniques. The micelles produced were relatively stable with an initial drop in size, probably caused by evaporation of residual solvent and/or release of fluconazole into the bulk liquid. The preparation methods based on membrane and microfluidic micromixing were simple, effective and reproducible.

## Figures and Tables

**Table 1:** Number-average molecular weight, M_n_, polydispersity index, M_w_/M_n_ (M_w_ is a weight-average molecular weight), and hydrophilic fraction (fraction of hydrophilic block to total polymer) by number- and weight-average molecular weight, respectively, f_n_ and f_w_, of the synthesized copolymers, as determined by GPC.

Polymer	PEG/PCL Mole Ratio in the Feed Mixture	M_n_	M_w_	M_w_/M_n_	f_n_	f_w_
PEG-*b*-PCL (A)	1:1.5	9666	12,215	1.26	0.62	0.52
PEG-*b*-PCL (B)	1:2.0	13,070	17,371	1.33	0.46	0.36
PEG-*b*-PCL (C)	1:3.0	16,027	20,945	1.31	0.37	0.30

**Table 2:** The Diameter and Polydispersity index (PDI) of micelles formed using different copolymers, fabrication techniques and organic solvents. The copolymer concentration in the organic phase used for preparation of drug-free micelles was 1 mg·mL^−1^. The organic phase used for preparation of drug-loaded micelles contained 0.6 mg·mL^−1^ of fluconazole and 1.5 mg·mL^−1^ of the copolymer. In membrane experiments, the pore size was 20 μm, the organic phase flow rate was 9.62 mL·min^−1^ (the flux was 600 L m^2^h^−1^), the organic phase volume was 5 mL, and the aqueous phase volume was 25 mL.

Organic Solvent	Technique		Drug-Free Micelles			Drug-Loaded Micelles		
			PEG-*b*-PCL (A)	PEG-*b*-PCL (B)	PEG-*b*-PCL (C)	PEG-*b*-PCL (A)	PEG-*b*-PCL (B)	PEG-*b*-PCL (C)
Acetone	Microfluidic	D (nm)	70 ± 9	55 ± 4	58 ± 8	87 ± 12	112 ± 11	84 ± 17
		PDI	0.177	0.193	0.120	0.205	0.213	0.203
	Membrane	D (nm)	53 ± 7	48 ± 7	53 ± 4	51 ± 7	63 ± 5	49 ± 0.49
		PDI	0.212	0.225	0.200	0.215	0.195	0.149
THF	Microfluidic	D (nm)	61 ± 15	53 ± 9	54 ± 11			
		PDI	0.264	0.204	0.236			
	Membrane	D (nm)	46 ± 7	41 ± 8	48 ± 3			
		PDI	0.261	0.193	0.206			

**Table 3:** The mean diameter of the drug-free micelles prepared using three copolymers dissolved in acetone from three different preparation techniques. The organic phase was 1 mg·mL^−1^ PEG-*b*-PCL in acetone.

Preparation Technique	PEG-*b*-PCL (A)		PEG-*b*-PCL (B)		PEG-*b*-PCL (C)	
	Diameter (nm)	PDI	Diameter (nm)	PDI	Diameter (nm)	PDI
Stirred tank without membrane	80 ± 6	0.205	83 ± 10	0.123	69	0.201
Microfluidic device	70 ± 9	0.177	55 ± 4	0.193	58 ± 8	0.120
Stirred tank with membrane	53 ± 8	0.212	48 ± 4	0.254	53 ± 5	0.200

**Table 4:** The mean diameter of micelles produced using membrane device as a function of drug concentration in the organic phase. The organic phase was 1 mg·mL^−1^ PEG-*b*-PCL (B) in acetone with designated drug content.

Drug Concentration in Organic Phase (mg·mL^−1^)	Diameter (nm)	PDI
0.50	57 ± 0.4	0.220
1.00	62 ± 4	0.195
2.50	74 ± 0.5	0.172

**Table 5:** HPLC derived measurements of encapsulated drug immediately after producing drug-loaded micelles (Day 0) and 48 h after producing micelles (Day 2) from the membrane device. The fluconazole content in the organic phase was 0.4 mg·mL^−1^ per 1 mg·mL^−1^ PEG-*b*-PCL (B) or PEG-*b*-PCL (C), respectively.

Polymer	Encapsulated Drug (mg·mL^−1^)	
	Day 0	Day 2
PEG-*b*-PCL (B)	0.23	0.17
PEG-*b*-PCL (C)	0.21	0.11

**Table 6:** The zeta potential of micelles formed by injecting 5 mL of the organic phase into 25 mL of water through the membrane. The organic phase for drug-free micelles was 1 mg·mL^−1^ of PEG-*b*-PCL in acetone. The organic phase for drug-loaded micelles was 1 mg·mL^−1^ of fluconazole per 1 mg·mL^−1^ of PEG-*b*-PCL in acetone.

Micelle Type	Zeta Potential (mV)		
	PEG-*b*-PCL (A)	PEG-*b*-PCL (B)	PEG-*b*-PCL (C)
Drug free	−11	−12	−12
Drug loaded	−7	−5	−6
